# Postural Changes on Heart Rate Variability among Older Population: A Preliminary Study

**DOI:** 10.1155/2021/6611479

**Published:** 2021-02-27

**Authors:** Warawoot Chuangchai, Wiraporn Pothisiri

**Affiliations:** College of Population Studies, Chulalongkorn University, Bangkok, Thailand

## Abstract

**Objective:**

This study aims to investigate an association between body postures and autonomic nervous system (ANS) responses through analysis of short-term heart rate variability (HRV) data obtained through electrocardiography.

**Methods:**

Forty older individuals were recruited to form the sample. HRV measurements were taken in three positions—sitting, supine, and standing—and compared.

**Results:**

Results demonstrated statistically significant differences in the HRV parameters used to examine the parasympathetic nervous system (PNS) and the sympathetic nervous system (SNS), specifically in the measurements obtained from the sitting position and the supine position (*P* < 0.001 for PNS and *P* = 0.011 for SNS). The differences in these parameters were, however, negligible between the sitting and the standing positions. Moreover, the ANS responses obtained in the sitting position were strongly and positively correlated with those in the standing position (*r* = 0.854 for PNS and *r* = 0.794 for SNS). These results suggested that the PNS and SNS parameters obtained while sitting were likely to be affected by orthostatic hypotension in much the same way as those in the standing position, as compared to the supine position.

**Conclusions:**

As such, sitting may not be the best position for older individuals in the assessment of their autonomic responses, whereas the supine position is recommended as the baseline posture in the old-age population. These findings are useful for future research in clinical settings that require accuracy in the ANS responses as determined by the HRV measurements.

## 1. Introduction

Aging is generally associated with a deterioration of the autonomic nervous system (ANS), which controls vital functions of the human body [1]. Declining ANS function causes an imbalance between the parasympathetic nervous system (PNS) and the sympathetic nervous system (SNS). Since ANS activities are linked with cardiac functions, the measurement of time variation between heartbeats—also known as the heart rate variability (HRV)—has been increasingly used to assess the condition of the ANS [2]. Electrocardiography (ECG) is a noninvasive technique that can detect the *R* wave in the QRS complex and calculate the time between *R* waves, or the *R*-*R* interval (*RR*), through measurements of HRV. A short-term (i.e., 5 minutes) test has been extensively used as a standard for HRV analysis [3]. The HRV test is typically conducted in sitting, supine, and standing positions [4]. The sitting and supine positions are frequently referred to as relaxed postures, while the standing position is commonly known as an active posture.

Previous studies have shown that HRV varies with a range of factors, including cardiac autonomic modulation [5, 6]. For older adults, the incidence of orthostatic hypertension is significantly correlated with variation in heart rates [7–9]. Orthostatic or postural hypotension is defined as a systolic blood pressure decrease of at least 20 mm Hg or a diastolic blood pressure decrease of at least 10 mm Hg within 3 minutes of standing up [10]. This condition is quite common for older people, but its prevalence can vary for different groups, with older community dwellers having the lowest rate, long-term care residents having the highest, and hospitalized older adults falling in between [11]. The evolution of cardiovascular control with age via several HRV markers has been reported extensively in the literature [12–15].

The tone in arteries, veins, and the heart is modulated by the SNS. Baroreceptors located primarily in the carotid arteries and aorta are very sensitive to changes in blood pressure. When the baroreceptors sense even the slightest drop in pressure, a coordinated increase in sympathetic outflow occurs. The peripheral resistance and the blood pressure are increased by the constriction of the arteries, which are affected by the increased heart rate (HR) and contractility [16]. These mechanisms are stabilized in maintaining blood pressure and perfusion. When the mechanisms are unable to balance the blood pressure, orthostatic hypotension may occur [17].

Orthostatic hypotension often occurs when one's position changes rapidly, and it may result in several symptoms, such as headache, blurred vision, and dizziness [18]. A change from a horizontal to a vertical posture shifts one's center of gravity. The rapid adjustment of the body to maintain blood pressure stimulates modulations of the ANS [19]. There is an increase in the SNS activity and a simultaneous decrease in the vagal nerve, or PNS, activity. Thus, the predominance of the ANS is shifted from the PNS to the SNS in a change from the supine position to the standing position [20].

Several HRV indices have been used to assess the variation of ANS modulations, for example, systolic arterial pressure, Shannon entropy, low-frequency power (LF), high-frequency power (HF), and LF/HF ratio [12–15]. Since the ANS is characterized by a dynamic balance between the PNS and the SNS activities—which originate via multiple HRV regulatory mechanisms, several indices of HRV can thus be used to monitor the ANS modulations. The PNS activity has been known to reduce the HR and increase the respiratory sinus arrhythmia (RSA) component. A larger mean RR is generally associated with a lower HR. The root mean square of successive differences (RMSSD) of RR is proportional to the RSA magnitude and also correlates with the Poincaré plot index of standard deviation 1 (SD1). On the other hand, the SNS activity increases the mean HR, Baevsky's stress index, and Poincaré plot index of SD2 [2, 21, 22]. In terms of the frequency-domain parameters, the LF (0.04–0.15 Hz) can be used to characterize the SNS response, while the HF (0.15–0.40 Hz) can be used to show the PNS response. In addition, the LF/HF ratio can be used to show the sympathovagal balance [3, 23]. However, in the event of normal breathing, particularly when the spontaneous respiratory rate is low—under 0.15 Hz or 9 breaths per minute, as normally found in older adults, the LF component could partly or even completely be overlapped by the RSA component. In this case, the LF and the LF/HF ratio can lead to an incorrect interpretation of the ANS response [24].

The current guidelines for recording HRV in a nonactivity stage recommend that the resting control position be used as the baseline posture for assessing an intervention [3, 25]. The resting control posture involves sitting upright with the knees at an angle of 90°, both feet flat on the floor, and both hands on thighs [25]. For older people, however, there is a possibility that the sitting position, even the resting control posture, could motivate orthostatic hypotension during the HRV measurement [26]. While most studies have focused on orthostatic hypotension induced in the standing position, much less information is available concerning other relaxed postures. The present study aims to fill the gap in the literature by investigating whether the ANS responses in older people were affected by orthostatic hypotension in the sitting, supine, and standing positions. Based on the results obtained, a recommendation is made on the baseline posture for measuring HRV with a minimal effect from orthostatic hypotension.

## 2. Materials and Methods

### 2.1. Participants

This study was undertaken at the Ayutthaya Social Welfare Development Center for Older Persons. Similar centers were established nationwide to promote the social participation of Thai older persons. One hundred and fifty individuals aged 60 years and older who were members of the center were randomly selected for initial screening. All potential candidates who reported to have no medical records of cardiovascular diseases, such as congestive heart failure, abnormal heart rhythm, stroke, and arrhythmia and were able to change their body postures without any assistance were invited to attend the orientation program in which all essential details of the project and the experimental procedures were explained. Among them, twelve potential candidates decided to withdraw from the study due to their busy schedule, resulting in the final sample size of 40 older participants.

### 2.2. Measurements

Demographic and health data of the participants, including sex, age, diseases, and daily drug treatments, were collected from their medical records, and official reports were obtained from the center. Each participant's weight and height were measured for the calculations of body mass index (BMI) and body fat. Systolic blood pressure (SBP) and diastolic blood pressure (DBP) were measured with a digital upper-arm blood pressure gauge. All of these measurements were conducted prior to the short-term HRV test.

The short-term HRV tests were conducted between 9 and 11 a.m. and between 2 and 5 p.m. in a quiet, private room. The room was illuminated with low ambient light from an artificial source and was maintained at a comfortable temperature of 25°C. The test was performed on one participant at a time in a sequence that was randomly assigned by drawing lots. The participants were asked to refrain from ingesting caffeine for at least 12 hours before the test and to wear comfortable shirts and shorts made of nonconductive materials. Before the test started, each participant was asked to sit back and relax, with bare feet, in a chair for 5 minutes. The skin of the right wrist and both ankles was gently cleaned with 70% isopropyl alcohol, after which three electrodes and a conductive gel were applied based on the two-lead method. The ECG measurements were taken with a sampling rate of 1,000 Hz using the PowerLab 26T data acquisition system (ADInstruments). During the course of the test, the participant was asked to wear the electrodes at all times.

Three positions were specified in a single run of the HRV test, in the following order: the sitting position, the supine position, and the standing position. The participant was instructed to have no conversations, to keep both eyes open to refrain from falling asleep, to try not to move the body, and to breathe spontaneously during the test. For the sitting position, each participant was instructed to sit in a chair with no armrests in the resting control posture for 10 minutes. The seat of the chair was 40 cm wide and 42.5 cm long and located 42.5 cm above the floor. The backrest was 35 cm wide and 50 cm high and positioned at an angle of 105° to the seat. After sitting for 10 minutes, the participant was asked to take a few short steps from the chair and up a medical two-step stool and then to lie down on the table in the supine position for another 10 minutes. In this position, the participant's arms rested at the side of the body while the face, torso, forearms, and palms were facing up. Feet were loose and aligned with the shoulders. The examination table was 80 cm wide, 190 cm long, and 75 cm high, and it had a small, flat pillow. Next, the participant was invited to come down from the examination table without any assistance and to stay in a standing posture for 5 minutes. Each of the participants was instructed to stand up following the same procedure: by tilting the trunk to the left, pushing the body to an upright sitting position, putting the left foot and then the right foot on the floor, resting for 5 seconds, and finally standing up for 5 minutes. The HRV data were recorded using the LabChart software for a total duration of 15 minutes, with 5 minutes in each posture, by disregarding the first 5 minutes of the sitting and the supine positions to avoid possible impacts from extraneous factors.

### 2.3. Statistical Analysis

Histograms and normal probability plots were utilized to test the normality of the data. The demographic and health characteristics of the participants were described with means and standard deviations. The HRV data were analyzed by using the Kubios software [27], 3.3.1 Standard version. The subparameters computed for the PNS index included mean RR, RMSSD and SD1. The subparameters computed for the SNS index were mean HR, stress index and SD2. The frequency-domain parameters—i.e., LF, HF, and LF/HF ratio in normalized units—were also computed. To deal with the issue of multiple comparisons of the measurements obtained from the three different positions, the analysis of variance (ANOVA) with repeated measures was conducted with the Wilks' lambda test. Subsequently, the paired *t*-test was performed on all of the computed indices, and the corresponding subparameters between any pair of the three postures taken in the HRV measurements. Relationships between the sitting and the standing positions were further examined using the Pearson correlation coefficient (*r*). All statistical analyses were performed with the SPSS statistics 22 software. A power calculation in matched pairs was 0.87 at a significance level of 0.05 (two-tailed), as computed by the *G* *∗* Power software 3.1.9.3 version.

## 3. Results

Table 1 presents means and standard deviations for the demographic and health characteristics of the sample. The sample consists disproportionately of females and middle-old age individuals with an average age of 76.4 years. The mean weight and height of the sample are 53.45 kg and 152.53 cm, respectively. The BMI and body fat are on average 22.98 kg/m^2^ and 37.35%, respectively. The BMI is in a normal range for Asians, as indicated in other studies (21–27.4), as well as by the WHO (18.5–23) [28]. The average number of illnesses is 1.9. The participants take on average 1.4 types of medicine per day. As prevalent among the general population aged 50 years and older [29, 30], the study sample has an average SBP of 145.13 mm Hg and an average DBP of 68.48 mm Hg, indicating isolated systolic hypertension, according to the guidelines for Chinese older adults [31].

Table 2 reports the computed values of means, along with the corresponding standard deviations of the PNS and SNS indices, the repeated measures ANOVA results for the three positions examined, and the paired *t*-test results between each pairing of these three positions—i.e., sitting-supine (ST-SP), supine-standing (SP-SD), and sitting-standing (ST-SD). Statistically significant differences were observed for both indices among the sitting, supine, and standing postures with *P* < 0.001 for the PNS index and *P* = 0.016 for the SNS index. In addition, there were statistically significant differences between the sitting and the supine positions, as well as between the supine and the standing positions, with *P* < 0.001 for the PNS index and *P* = 0.011 for the SNS index, respectively. Meanwhile, for each of the two indices, the discrepancy between the sitting position and the standing position was not statistically significant.

Table 3 shows the means, standard deviations, repeated measures ANOVA results, and the paired *t*-test results obtained for each of the computed subparameters of PNS and SNS. The repeated measures ANOVA results showed statistically significant differences among the three investigated positions in the mean RR and the mean HR with *P* < 0.001, while the differences were not significant for other PNS and SNS parameters. A closer look at the paired *t*-test results showed that, for PNS, the differences in the mean RR between the sitting and the supine positions, and between the supine and the standing positions were statistically significant, with *P* < 0.001, while the negligible difference was observed between the sitting and the standing positions. The paired *t*-test results also showed no statistically significant differences in RMSSD and SD1 for any pair of the positions examined. In terms of SNS, the only statistically significant differences were observed in the mean HR between the sitting and the supine positions, and between the supine and the standing positions, with *P* < 0.001.

Table 4 shows the means, standard deviations, repeated measures ANOVA results, and the paired *t*-test results obtained for each of the HRV markers in the frequency domain. Based on the repeated measures ANOVA results, the differences among the three positions were statistically significant only for the LF and the HF, with *P* *=* 0.004. For the paired *t*-test results, the differences in the LF and the HF between the supine and the standing positions, as well as that in the LF/HF ratio, were statistically significant with *P* = 0.001 and *P* = 0.022, respectively. Conversely, no significant differences in these HRV markers were found between sitting and the other two positions.

**Figure 1:**
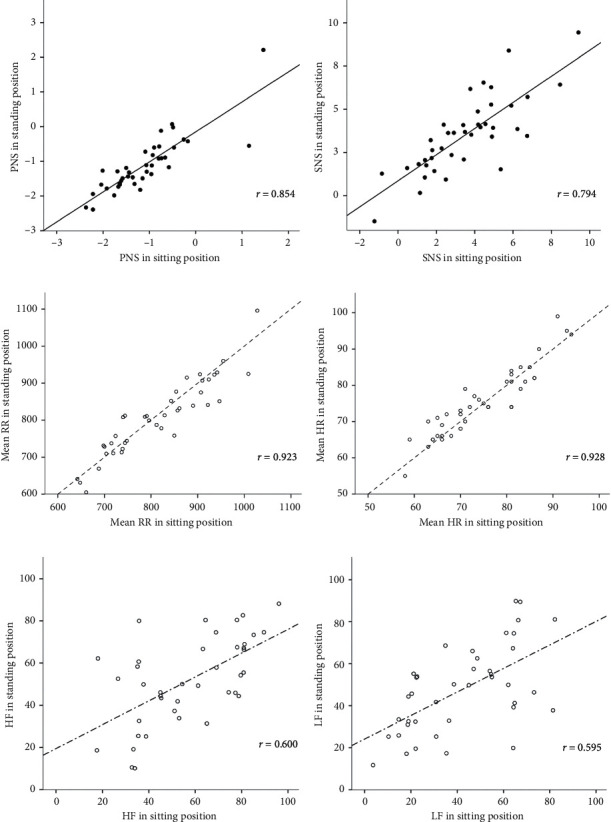
Pearson correlation coefficients between sitting and standing positions. (a) PNS between sitting and standing positions. (b) SNS between sitting and standing positions. (c) Mean RR between sitting and standing positions. (d) Mean HR between sitting and standing positions. (e) HF between sitting and standing positions. (f) LF between sitting and standing positions.

The Pearson correlation coefficients between the sitting position and the standing position are illustrated in Figure 1. Correlations between the PNS and SNS indices obtained from the two positions are represented by solid lines in the first and second rows, with the computed *r* values of 0.854 and 0.794, respectively. Similar relationships were observed for the mean RR and the mean HR, as shown by dashed lines in the third and fourth rows, with the computed *r* values of 0.923 and 0.928, respectively. These coefficients showed that both the PNS and the SNS index and the corresponding subparameters, obtained from the sitting and the standing positions, were strongly and positively correlated. For the frequency-domain parameters, as shown by the dashed-dotted lines in the fifth and sixth rows, the HF and the LF obtained from the sitting and the standing positions were found to be positively correlated only moderately, with the computed *r* values of 0.600 and 0.595, respectively.

## 4. Discussion

As previously stated, the current study is aimed at expanding the literature in two significant ways. First, while existing studies have adopted the sitting position as the baseline posture for HRV measurements, the current study examines a potential effect of orthostatic hypotension on the HRV data taken while sitting, even in the resting control posture. Second, by comparing several parameters of PNS and SNS collected in the sitting position to those obtained in the supine and the standing positions, it is possible to identify an optimal posture with a minimal effect from orthostatic hypotension for use as the baseline posture for older individuals in the assessment of their autonomic responses.

Our results showed that the ANS responses obtained from the HRV measurements taken in the sitting position were strongly and positively correlated with those taken in the standing position, whereas the only statistically significant differences in these responses were identified between the supine position and the other two postures. The statistical differences could also be detected in the HRV markers in the frequency domain, but only between the supine and the standing positions. These results were in line with the literature in which similarity between HRV markers obtained during supine and semisitting was also reported based on a modified head-up tilt test [32].

The results implied that the ANS responses obtained in the sitting position were likely to be affected by orthostatic hypotension in much the same way as those obtained in the standing position. Therefore, sitting—even in the resting control posture—may not be the best position for the assessment of HRV in older people, as compared with the supine position. Even though it has been used only for sleep studies [25], we recommend the supine position as the most relaxed position for use as the baseline posture in the HRV measurements for older people. To the best of our knowledge, this study was the first to explore such an association between the ANS responses and different postures among older Thais through the analysis of HRV.

The results obtained from the current study have potentially raised a crucial concern on the current practice of using sitting in the resting control posture as the baseline position for HRV measurements. In the literature, the potential effect of orthostatic hypotension induced in the sitting position has also been reported among aging hospitalized people [33], while such an impact is less pronounced in younger populations [34, 35]. For young adults and children, the results on the impact of postural changes on orthostatic hypotension are rather mixed, with some studies reporting differences in the autonomic responses between the sitting and the supine positions [36–38] and other studies showing no difference between the two positions [35, 39].

Even though standardized procedures and tools were employed, with no health-related issues reported, the current study had some limitations. The sample was unbalanced in terms of sex, whereas previous studies indicated gender differences in ANS responses to postural changes [40–42]. Furthermore, during the pilot stage, an electronic metronome rhythm was used to control the breathing pattern of the participants, who noted among their subjective stresses discomfort at relaxing with sound control. Since this was an influential factor in inducing stress, respiratory control was excluded in the final stage. Due to time restrictions imposed by the center, the experiments had to be conducted only in the morning and in the evening. The difference in time of day may have affected the ANS results through the variation of circadian rhythms. Moreover, further studies are needed to identify modulations of the ANS responses with respect to postural changes in older people with autonomic-related dysfunctions, such as orthostatic hypotension, Parkinson's disease, or dysautonomia. In addition, other body postures, such as prone, lateral recumbent, or semi-Fowler's positions, and other measuring protocols, such as head-up tilt test or passive tilt test, should also be investigated.

## 5. Conclusions

Through the analysis of HRV data obtained from a sample of 40 older Thais in three different positions, this study has identified the effect of orthostatic hypotension on the ANS responses in the sitting position, even in the resting control posture, and it has shown the effect to be similar to that in the standing position. As such, sitting may not be the most relaxed position for measuring HRV in the old-age population. The current study suggests the supine position, instead of the resting control position, as the baseline posture in HRV measurements for older individuals. The results obtained by this study are beneficial for future research in clinical settings that requires accuracy in the HRV measurements.

## Figures and Tables

**Table 1:** Demographic and health characteristics of the sample (*N* = 40).

Characteristics	Mean (standard deviation)
Female (%)	77.50
Age (years)	76.40 (7.49)
Weight (kg)	53.45 (7.58)
Height (cm)	152.53 (7.96)
BMI (kg/m^2^)	22.98 (2.92)
Body fat (%)	37.35 (6.35)
Illnesses (counts)	1.90 (1.17)
Daily medicine intake (types)	1.37 (1.39)
SBP (mm Hg)	145.13 (20.54)
DBP (mm Hg)	68.48 (11.08)

**Table 2:** Descriptive statistics, repeated measures ANOVA, and paired *t*-test for ANS indices.

ANS index	Mean (standard deviation)			Repeated measures ANOVA results	Paired *t*-test results		
	Sitting (ST)	Supine (SP)	Standing (SD)		ST-SP	SP-SD	ST-SD
PNS index	−1.11 (0.79)	−0.84 (0.77)	−1.11 (0.80)	^*∗∗∗*^	^*∗∗∗*^	^*∗∗∗*^	NS
SNS index	3.53 (2.30)	2.91 (2.17)	3.53 (2.19)	^*∗*^	^*∗*^	^*∗*^	NS

*Note.* Symbols ^*∗*^, ^*∗∗*^, and ^*∗∗∗*^ denote significant levels at 0.05, 0.01, and 0.001, respectively; NS = no significance.

**Table 3:** Descriptive statistics, repeated measures ANOVA, and paired *t*-test for PNS and SNS subparameters.

Subparameter	Mean (standard deviation)			Repeated measures ANOVA results	Paired *t*-test results		
	Sitting (ST)	Supine (SP)	Standing (SD)		ST-SP	SP-SD	ST-SD
PNS							
Mean RR (ms)	815.13 (103.16)	865.30 (113.05)	807.30 (100.92)	^*∗∗∗*^	^*∗∗∗*^	^*∗∗∗*^	NS
RMSSD (ms)	14.53 (12.47)	15.79 (9.52)	16.23 (14.91)	NS	NS	NS	NS
SD1 (ms)	10.31 (8.84)	11.18 (6.74)	11.50 (10.56)	NS	NS	NS	NS
SNS							
Mean HR (bpm)	74.73 (9.60)	70.55 (9.35)	75.48 (9.48)	^*∗∗∗*^	^*∗∗∗*^	^*∗∗∗*^	NS
Stress index	29.05 (11.46)	26.90 (10.74)	28.64 (11.00)	NS	NS	NS	NS
SD2 (ms)	12.86 (8.28)	13.64 (5.73)	15.69 (13.00)	NS	NS	NS	NS

*Note.* ^*∗*^, ^*∗∗*^, and ^*∗∗∗*^ denote significant levels at 0.05, 0.01, and 0.001, respectively; NS = no significance.

**Table 4:** Descriptive statistics, repeated measures ANOVA, and paired *t*-test for HRV markers in the frequency domain.

HRV markers in frequency domain	Mean (standard deviation)			Repeated measures ANOVA results	Paired *t*-test results		
	Sitting (ST)	Supine (SP)	Standing (SD)		ST-SP	SP-SD	ST-SD
LF	42.19 (21.94)	39.41 (17.57)	47.78 (20.62)	^*∗∗*^	NS	^*∗∗*^	NS
HF	57.43 (21.76)	60.34 (17.57)	51.96 (20.52)	^*∗∗*^	NS	^*∗∗*^	NS
LF/HF ratio	1.09 (1.08)	0.85 (0.74)	1.53 (1.94)	NS	NS	^*∗*^	NS

*Note.* Symbols ^*∗*^, ^*∗∗*^, and ^*∗∗∗*^ denote significant levels at 0.05, 0.01, and 0.001, respectively; NS = no significance.

## Data Availability

Data employed in this study are not publicly available due to the restrictions imposed by data providers and the ethical approval that governs the fieldwork. Access to data would only be granted upon request. For further inquiries, please contact via warawoot.c@chula.ac.th.
